# Variability over 1 Week in the Urinary Concentrations of Metabolites of Diethyl Phthalate and Di(2-Ethylhexyl) Phthalate among Eight Adults: An Observational Study

**DOI:** 10.1289/ehp.1002231

**Published:** 2010-08-25

**Authors:** James L. Preau, Lee-Yang Wong, Manori J. Silva, Larry L. Needham, Antonia M. Calafat

**Affiliations:** National Center for Environmental Health, Centers for Disease Control and Prevention, Atlanta, Georgia, USA

**Keywords:** biomonitoring, DEHP, DEP, exposure, human, phthalates, urine, variability

## Abstract

**Background:**

Phthalates are metabolized and eliminated in urine within hours after exposure. Several reports suggest that concentrations of phthalate metabolites in a spot urine sample can provide a reliable estimation of exposure to phthalates for up to several months.

**Objectives:**

We examined inter- and intraperson and inter- and intraday variability in the concentrations of monoethyl phthalate (MEP), the major metabolite of diethyl phthalate, commonly used in personal care products, and mono(2-ethyl-5-hydroxyhexyl) phthalate (MEHHP), a metabolite of di(2-ethylhexyl) phthalate (DEHP), a polyvinyl chloride plasticizer of which diet is the principal exposure source, among eight adults who collected all urine voids (average, 7.6 samples/person/day) for 1 week.

**Methods:**

We analyzed the urine samples using online solid-phase extraction coupled to isotope dilution–high-performance liquid chromatography–tandem mass spectrometry.

**Results:**

Regardless of the type of void (spot, first morning, 24-hr collection), for MEP, interperson variability in concentrations accounted for > 75% of the total variance. By contrast, for MEHHP, within-person variability was the main contributor (69–83%) of the total variance. Furthermore, we observed considerable intraday variability in the concentrations of spot samples for MEHHP (51%) and MEP (21%).

**Conclusions:**

MEP and MEHHP urinary concentrations varied considerably during 1 week, but the main contributors to the total variance differed (interday variability, MEHHP; interperson variability, MEP) regardless of the sampling strategy (spot, first morning, 24-hr collection). The nature of the exposure (diet vs. other lifestyle factors) and timing of urine sampling to evaluate exposure to phthalates should be considered. For DEHP and phthalates to which people are mostly exposed through diet, collecting 24-hr voids for only 1 day may not be advantageous compared with multiple spot collections. When collecting multiple spot urine samples, changing the time of collection may provide the most complete approach to assess exposure to diverse phthalates.

Phthalates are high-production-volume chemicals. Some phthalates make plastics pliable and may be used in vinyl flooring, medical devices, wall coverings, toys, and food containers. Other phthalates are often found in personal care products (e.g., cosmetics, lotions, perfumes) and in the coatings of some medications (David et al. 2001; Koch and Calafat 2009; Schettler 2006). The ubiquitous use of phthalates results in widespread human exposure [Centers for Disease Control and Prevention (CDC) 2010]. In humans, phthalates are rapidly metabolized to their corresponding hydrolytic monoesters, which can be further transformed to oxidative products, conjugated, and eliminated (Koch and Calafat 2009).

Urinary phthalate metabolite concentrations represent an integrative measure of exposure to phthalates from multiple sources and routes. Therefore, phthalate metabolites have been used extensively as exposure biomarkers (Koch and Calafat 2009). However, personal exposure to phthalates is likely to vary over time as a result of changes in the use of personal care products, diet, or daily activities. Although the urinary concentrations of phthalate metabolites can be used to accurately measure the exposure of a person at a single point in time, because of the short elimination half-life (t_1/2_) of phthalates (i.e., a few hours) (Koch and Calafat 2009), determining exposure over weeks or months may require multiple measurements. Therefore, information on the temporal variability of urinary concentrations of phthalate metabolites is needed to optimize the design of exposure assessment in epidemiologic studies.

Several studies have shown that the concentrations of phthalate metabolites in a single urine sample can provide a reliable ranking to classify a person’s exposure to phthalates for up to several months, although some metabolites display more temporal variability than others (Adibi et al. 2008; Fromme et al. 2007; Hauser et al. 2004; Hoppin et al. 2002; Peck et al. 2010; Teitelbaum et al. 2008). These reports suggest that urinary concentrations of phthalate metabolites in serial samples vary over periods of time. However, to date, no studies have addressed either intraday variability or the variability of spot samples, first morning voids, and 24-hr collections obtained from the same person.

We report the variability over a period of 1 week in phthalate exposure by using the metabolites of two example phthalates, diethyl phthalate (DEP) and di(2-ethylhexyl) phthalate (DEHP). We chose these phthalates because of their relatively short elimination t_1/2_ but differing main exposure pathways and metabolism. The main exposure pathway to DEP is dermal absorption from the use of personal care products that contain DEP and, to a lesser extent, ingestion and inhalation (Api 2001). By contrast, ingestion of food is the likeliest source of exposure to DEHP (Kavlock et al. 2006). DEP is a low-molecular-weight phthalate metabolized mainly to its hydrolytic monoester, monoethyl phthalate (MEP), before urinary excretion. Conversely, DEHP is a high-molecular-weight phthalate that is also first hydrolyzed to its hydrolytic monoester but undergoes further metabolism, which results in numerous products, including the oxidative metabolite mono(2-ethyl-5-hydroxyhexyl) phthalate (MEHHP).

We present the variability of urinary concentrations of MEP and MEHHP in eight adults over 7 consecutive days. This study provides data to help elucidate patterns of within- and between-person and of within- and between-day variability. Furthermore, this study is the first to provide information of the variance apportionment of the concentrations of phthalate metabolites by person, day, and time of day for spot, first morning, and 24-hr void urine samples collected from the same person.

## Methods

### Study design

In 2005, eight adults were recruited to participate in a study designed to examine the temporal variability in the urinary concentrations of several polycyclic aromatic hydrocarbon (PAH) metabolites (Li et al. 2010). The study volunteers (four males and four females) were healthy, nonsmoking CDC employees living in the metropolitan Atlanta, Georgia, area, ranging in age from 25 to 59 years, with no documented occupational exposure to PAH or phthalates [see Supplemental Material, Table 1 (doi:10.1289/ehp.1002231)]. The institutional review board of CDC approved this study, and all participants provided written informed consent.

During a 1-week study period in October–November 2005 [see Supplemental Material, Table 1 (doi:10.1289/ehp.1002231)], each person, while engaged in his or her normal daily activities, collected all urine voids throughout the day and night in a commercial nonvinyl plastic specimen collection container. After collection, the participants recorded the urine volume and time of the void, decanted an approximately 50-mL aliquot of the urine to a prelabeled, sterile, 4-oz polypropylene/polyethylene urine collection cup, stored the cup in an ice cooler containing frozen ice packs, and discarded the remainder of the urine. The urine samples were retrieved from each participant daily (or after the weekend), aliquoted into polypropylene cryovials or glass jars, and frozen at −70°C until analysis. Participants also noted day and time of missed collections and recorded detailed information on their diet, driving, and outdoor activities during the week of the study.

### Phthalate metabolite measures

The approach for determining the urinary concentrations of phthalate metabolites has been described in detail (Kato et al. 2005). Briefly, the analytical method involved enzymatic deconjugation of the phthalate metabolites from the glucuronidated form, followed by online solid-phase extraction, separation with high-performance liquid chromatography, and detection and quantitation by isotope-dilution tandem mass spectrometry. Analytical standards, quality control (QC) materials (prepared from spiked pooled urine), and reagent blank samples were included in each batch along with study samples. The QC concentrations—averaged to obtain one measurement of high-concentration QC and one of low-concentration QC for each batch—were evaluated by using standard statistical probability rules (Caudill et al. 2008). With the analytical method used, we can obtain data for up to 16 phthalate metabolites. However, for the statistical analysis, we considered only MEP and MEHHP. Creatinine, used to correct for the dilution of the urine, was measured at CDC by using an enzymatic reaction on a Roche Hitachi 912 chemistry analyzer (Hitachi, Pleasanton, California).

### Statistical analysis

Statistical analyses were carried out using SAS version 9.2 (SAS Institute Inc., Cary, NC). Concentrations below the limit of detection (LOD) (MEP: 0.4 μg/L; MEHHP: 0.32 μg/L) were replaced with the LOD divided by the square root of 2 (Hornung and Reed 1990). The urinary metabolite concentrations followed a log-normal distribution; therefore, all data were log_10_-transformed before statistical analysis. First morning voids were defined as the first sample collected from each person at or after 0500 hours each day. A simulated 24-hr void concentration was calculated as the volume-weighted average of all specimens collected by an individual during a 24-hr period starting at midnight.

To assess the impact of creatinine adjustment to the total variance when exposure is categorized from the urinary concentrations of spot samples, we built three different models: *a*) without creatinine correction for urinary dilution (unadjusted); *b*) using creatinine-corrected concentrations (creatinine corrected); and *c*) including creatinine as a model covariate (creatinine adjusted). We ranked these models based on their Akaike information criterion (AIC) values (the lower the AIC, the better the model). To assess the temporal variability in phthalate concentrations, we calculated intraclass correlation coefficients (ICCs) for the three collections (spot, first morning, and simulated 24-hr voids). The ICC indicates the temporal reproducibility of repeated measures and is computed by dividing the estimate of the between-person variance by the estimated total variance. ICC ranges from 0 (poor reliability) to 1 (high reliability). To generate the variance component in the calculation of the ICCs, we used the three-level unconditional (intercept only) random-effect model. Level 1 is the time (*i*), which is nested within the day (level 2, *j* = 7), which is nested within the participants (level 3, *k* = 8). The only fixed effect was the grand mean of the intercept. The model equation was *Y_ijk_* = (*Y*_000_) + (*V*_00_*_k_* + *U*_0_*_jk_* + γ*_ijk_*), where *Y_ijk_* is the log_10_ (metabolite concentration) for participant *k* on day *j* at time *i. Y*_000_ is the grand mean (intercept), and *V*_00_*_k_*, *U*_0_*_jk_*, and γ*_ijk_* are the random errors for level 3, level 2, and level 1 residual, respectively.

We categorized the time of sample collection as morning (after midnight–1159 hours), afternoon (1200–1800 hours), and evening (1801–2359 hours), and calculated the geometric mean (GM) concentration for each collection time. We examined the association between time of sample collection and the urinary concentrations in a one-way mixed model.

## Results

We analyzed a total of 427 urine samples collected within the 7-day period from eight adult participants [see Supplemental Material, Table 1 (doi:10.1289/ehp.1002231)]. MEP was detected in all except one sample. MEHHP was detected in every sample collected from four of the eight participants; for the remaining four persons, MEHHP detection frequency ranged from 95 to 98%. The GM, median, and interquartile concentrations from all samples, first morning voids, and simulated 24-hr voids are shown in Table 1.

**Figure 1:**
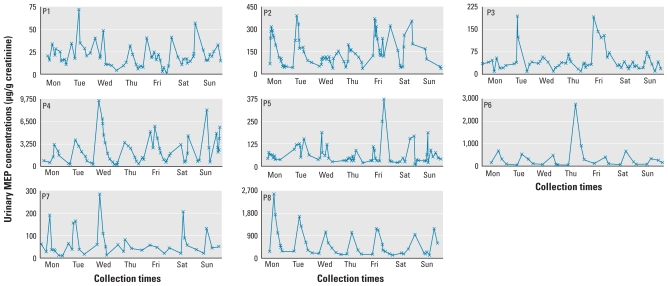
Creatinine-corrected concentrations of MEP (μg/g creatinine) for all study participants (P1–P8) during 1 week.

**Figure 2:**
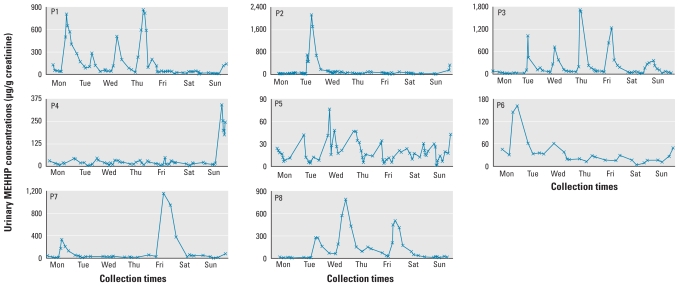
Creatinine-corrected concentrations of MEHHP (μg/g creatinine) for all study participants (P1–P8) during 1 week.

Throughout the study week, urinary concentrations varied by up to two (MEP) and three (MEHHP) orders of magnitude. Of interest, concentrations of MEP (Table 2, Figure 1) and MEHHP (Table 3, Figure 2) for each person varied considerably throughout the day. The average intraperson coefficient of variation (CV) of creatinine-corrected MEP concentrations in the spot urine samples collected throughout the week was 92% and ranged from 62% (participant P1) to 157% (participant P6). Similarly, for creatinine-corrected MEHHP concentrations, intraperson CVs averaged 161% and ranged from 74% (participant P5) to 263% (participant P2).

Furthermore, when the urinary concentrations of each study participant were plotted over the week, participants P6 and P8 showed a pronounced cyclic pattern of MEP measurements for every day of the week. Participant P4 also had a daily MEP pattern, albeit only for 4–5 days, but for the other participants (P1, P2, P3, P5, P7; Figure 1), a clear visual pattern was not as evident. A less-pronounced MEHHP daily cyclic pattern was observed only for participants P1 and P3 (Figure 2). We observed similar patterns, although with the differences somewhat less pronounced for each participant, for the log_10_-transformed concentrations [see Supplemental Material, Figures 1–2 (doi:10.1289/ehp.1002231)].

In Table 4, we present the variance components when the MEP and MEHHP concentrations of the spot urine samples are included as *a*) unadjusted (i.e., in micrograms per liter without accounting for dilution), *b*) creatinine corrected (i.e., in micrograms per gram creatinine accounting for dilution), or *c*) creatinine adjusted (by including creatinine as a model covariate). Independent of the dilution treatment, for MEP, the between-person variance contributed 64–77% of the total variance. By contrast, for MEHHP, total within-person variance (between days + within days) was predominant, accounting for approximately 80% of the total variance. However, for both phthalate metabolites, both the creatinine-corrected and creatinine-adjusted models yielded almost identical AIC values that, in turn, were much smaller than the AIC values obtained from models of the unadjusted concentrations. These results suggest that differences in urine dilution explained some of the variance in the metabolite concentrations, because the model fits improved when we accounted for dilution by correcting or adjusting for creatinine concentration. Therefore, for all subsequent variance calculations, we used the creatinine-corrected concentrations, because the simulated 24-hr voids data cannot be creatinine adjusted.

MEP is the hydrolytic and primary metabolite of DEP, whereas MEHHP is an oxidative metabolite of DEHP; MEHHP elimination t_1/2_ is about 3–4 times longer than that of MEP. To explore whether some of the observed differences in the variance components are related to differences in elimination t_1/2_ of MEP and MEHHP, we calculated the inter- and within-person variability for the hydrolytic metabolite of DEHP, mono(2-ethylhexyl) phthalate (MEHP), which has a t_1/2_ comparable with that of MEP. The MEHP and MEHHP results were similar [see Supplemental Material, Table 2 (doi:10.1289/ehp.1002231)].

The contributions to the total variance of the creatinine-corrected concentrations between and within persons observed for all types of collections for the study week are shown in Table 5. The ICCs of between-person concentrations were 0.25 (MEHHP) and 0.91 (MEP) for first morning voids, 0.17 (MEHHP) and 0.77 (MEP) for spot samples, and 0.31 (MEHHP) and 0.94 (MEP) for simulated 24-hr voids. For MEP, the between-person variance (ranging from 77% to 94%) was much higher than the total within-person variance (between days + within days) (6–23%). Conversely, for MEHHP, the total within-person variance contribution (69–83%) was higher than the between-person variance contribution (17–31%). Of interest, for both analytes for all the spot samples collected, the within-day variance was higher than the between-day variance contribution for each participant. For the spot samples, 51% (for MEHHP) and 21% (for MEP) of the variability was explained by within-day variation in individuals, rather than by variation between participants or between days in individuals. To further explore the potential influence of the time of collection on within-day variability, we categorized the spot samples as morning, afternoon, and evening. For MEP, the GM concentrations of samples collected in the morning (73 μg/L) and afternoon (72 μg/L) were significantly higher (*p* < 0.01) than in those collected in the evening (52.8 μg/L). By contrast, for MEHHP, the GM concentration of samples collected in the evening (33.2 μg/L) was significantly higher (*p* < 0.01) than in samples collected in the morning (18.7 μg/L) or in the afternoon (18.1 μg/L).

## Discussion

As expected from the ubiquitous use of phthalates in modern societies, our data suggest widespread human exposure among this adult study population. The high detection frequency and concentrations were within the ranges reported for the adult U.S. general population from the National Health and Nutrition Examination Survey (NHANES) 2005–2006 (CDC 2010).

Six previous studies have evaluated the temporal variability of phthalate metabolites in various populations over periods ranging from days to months (Adibi et al. 2008; Fromme et al. 2007; Hauser et al. 2004; Hoppin et al. 2002; Peck et al. 2010; Teitelbaum et al. 2008). In three of these studies, researchers assessed the agreement of phthalate metabolite concentrations by using first morning urine samples collected from 46 African-American women on 2 consecutive days (Hoppin et al. 2002), from 50 German men and women 14–60 years of age during 8 consecutive days (Fromme et al. 2007), and from 25 Hmong women who provided up to three samples over approximately 30 days (Peck et al. 2010). In the other studies, researchers evaluated the variability of phthalate metabolite concentrations in multiple spot urine samples collected from 28 Dominican and African-American women who provided two to four samples over 6 weeks during their last trimester of pregnancy (Adibi et al. 2008), from 11 men who provided nine samples each over 3 months (Hauser et al. 2004), and from 35 Hispanic and African-American children 6–10 years of age who collected two to seven samples over 6 months (Teitelbaum et al. 2008). Except for one study (Fromme et al. 2007), all others assessed the variability of MEP, although two older reports (Hauser et al. 2004; Hoppin et al. 2002) did not assess the variability of MEHHP and other DEHP oxidative metabolites.

In agreement with the ICCs reported previously for MEHHP urinary concentrations of first morning voids over 8 days to ~ 1 month (Fromme et al. 2007; Peck et al. 2010) and of spot samples collected within 6 weeks to ~ 6 months (Adibi et al. 2008; Teitelbaum et al. 2008), we found that between-person MEHHP creatinine-corrected concentrations for our study population varied considerably over 7 consecutive days (ICC = 0.25 for first morning voids; ICC = 0.17 for spot samples). By contrast, we found a low variability of between-person creatinine-corrected concentrations of MEP during the same time period (ICC = 0.91 for first morning voids; ICC = 0.77 for spot samples). Other studies have also reported moderate (ICC = ~ 0.6) reproducibility in MEP urinary measures (Hauser et al. 2004; Hoppin et al. 2002; Peck et al. 2010).

In this study, the largest contribution of the total variance of MEP urinary concentrations in spot samples was the between-person variability (77%). Throughout the day, the average person’s variance was also considerable (21%), but the average person’s between-day variance was rather low (2%). Similarly, the largest percentage of total variance in MEP concentrations from first morning and 24-hr voids was also the variation between each person (91% and 94%, respectively).

DEP exposure is largely associated with the use of personal care products (Api 2001; Berman et al. 2009; Duty et al. 2005; Houlihan et al. 2002; Hubinger and Havery 2006; Koo and Lee 2004; Sathyanarayana et al. 2008; Schettler 2006). The large between-person variability of MEP urinary concentrations we observed among this group of adults is likely related to the fact that different people use different types and combinations of personal care products. On the other hand, the fact that the persons examined had a large variation in MEP urinary concentrations throughout a given day, but very small variation between days, may be related to their regular use of personal care products. We speculate that people typically use the same personal care products at similar times in their daily routines and that individuals also tend to apply personal care products in similar amounts and frequency every day. Furthermore, the regular use of personal care products at similar times every day and the short DEP elimination t_1/2_ could result in MEP being excreted every day at similarly spaced times. This behavioral use of DEP-containing personal care products may also explain the appearance of a cyclic pattern in MEP urinary concentration in the persons with the largest concentrations of MEP during the study week. Of interest, the cyclic pattern was particularly evident during the work week but seemed to change for many participants over the weekend.

Unlike MEP, the largest variation of MEHHP urinary concentrations in spot samples was related to the variation of each person throughout the day (51%). The within-person variability between days was also considerable (32%) and about twice the variation attributed to differences between persons (17%). Likewise, the largest contributor to the total variance of MEHHP concentrations in first morning and 24-hr urine voids was individual variability from day to day (75% and 69%, respectively). We obtained similar results for MEHP, the DEHP hydrolytic metabolite, even though MEHHP elimination t_1/2_ is 3–4 times longer than of MEHP, suggesting that the main factors affecting the observed variance for MEHHP and MEHP concentrations are similar. Exposure to DEHP, the MEHHP precursor in the body, is largely associated with the consumption of food (Kavlock et al. 2006). Not only do diets vary from person to person, but an individual’s food consumption typically changes from day to day. Consistent with this, we did not observe clear daily patterns in MEHHP urinary concentrations for most participants.

Our findings also suggest that, regardless of the type of sample collected (i.e., spot, first morning, and 24-hr voids), when diet is the likely main source of exposure (i.e., DEHP), interday variability is a main contributor to the total variance. By contrast, when routine daily use of a product is the main exposure source (i.e., DEP), interperson variability appears to be the main contributor to the total variance. However, age will have a strong impact in relation to exposure to environmental chemicals, including phthalates, because behavior and diet, among other factors, are likely contributors to exposure to these compounds. For example, for young children, particularly infants, diet may not be as diversified as it is for adults. The extent and patterns of use of personal care products among children and adults are also expected to differ. Therefore, some of the findings we report for this group of adults may not apply to children and other age groups. Furthermore, the number of study participants examined was rather small, although the reported MEP and MEHHP urinary concentrations fell within the NHANES reference ranges. For the above reasons, we recommend caution in the generalization of our findings to other populations.

Twenty-four hour urine specimens do not require a correction for the urine dilution, which is important because no consensus exists on the best method for conducting such adjustment (Adibi et al. 2008; Barr et al. 2005; Pearson et al. 2009). On the other hand, our findings suggest that collecting 24-hr samples for only 1 day could benefit studies designed to evaluate compounds to which people are mostly exposed through routine use of personal care products. Unfortunately, for many chemicals, the contribution to the total exposure from all potential sources is either variable or unknown. In addition, epidemiologic studies often evaluate exposure to a wide range of compounds (and their corresponding exposure sources). As a result, collecting 24-hr voids may not necessarily eliminate the potential for exposure misclassification, at least for some of the compounds examined. Therefore, when the population is sufficiently large, the spot-sampling approach may provide enough statistical power to adequately categorize exposure, particularly when samples are collected on multiple days.

One of the most important findings of this work is to show that, for a given person, the urinary concentrations of phthalate metabolites can change considerably throughout the day. Others have observed similar intraday variability in the urinary concentrations of other nonpersistent chemicals, such as PAH metabolites (Li et al. 2010). More important, even for the two metabolites we evaluated, the intraday changes went in opposite directions. For example, we found that the lowest MEP urinary concentrations, but the highest MEHHP concentrations, occurred in the evening. These findings suggest that sampling strategy should be one critical factor when designing epidemiologic studies that include biomonitoring measures of urine specimens. Very often these specimens are analyzed for more than one class of environmental chemicals. Therefore, when multiple collections of spot urine samples over a period of days to weeks or months are logistically and economically possible, specimens should be collected at different times of the day. Our data for MEP and MEHHP suggest that this approach would maximize the suitability of the urinary concentrations of the various target biomarkers to reflect temporal exposure to nonpersistent chemicals. However, when and how often urine samples are collected will depend not only on how reproducible the urinary concentrations are (i.e., relatively high ICC), but also on the target population, aims of the study, major route of uptake of the parent phthalate, and excretion t_1/2_ of its metabolites. For DEP, sampling around midday on any given day may be advantageous if exposure occurs mainly through the use of personal care products and if these products are applied in the morning, because MEP excretion t_1/2_ is 3–4 hr and peak excretion would be expected to occur around midday. Whether multiple sampling is needed for exposure assessment in specific situations (e.g., during pregnancy) will depend mainly on the intraperson variability at the sampling time (e.g., noon) and throughout the study period (e.g., 1 week). For DEHP, because of the strong influence of diet, the daily intraperson variability may be as high as the intraperson variability at one specific time of the day throughout the course of the study. When intraperson variability is unavoidable and highly independent of the sampling time, two potential approaches for conducting exposure assessment are as follows: *a*) use the mean or median urinary concentrations of all of the samples collected over a certain time period if multiple collections per person are possible, or *b*) if only one spot sample per person is available, use each individual concentration and provide estimates of upper and lower confidence intervals (CIs) based on results from all participants. For the latter, our findings might serve as a basis for setting such fixed upper and lower CIs of exposure, in particular, for epidemiologic studies where recruitment of participants has been completed and multiple sampling for exposure assessment is no longer possible.

Among participants of NHANES 1999–2000, variations have been reported in the distributions of urinary concentrations of MEP and other phthalate metabolites, depending on the time of day of sampling (Silva et al. 2004). In addition to this variability, we found that MEP urinary concentrations also differed by age and race/ethnicity (Silva et al. 2004). Although the nature of the exposure to phthalates and the short t_1/2_ of the phthalates will affect the urinary concentrations of phthalate metabolites on an individual basis, on a population basis, the range of concentrations observed in our study may represent an average exposure scenario. For example, MEP concentrations in the upper percentiles resulting from the collection of urine soon after DEP-related activity of an individual will likely be offset by a urinary concentration in the lower percentiles originating from another person who provided a sample shortly before conducting the same activity. In this study, the considerable variation in concentrations of urinary biomarkers of DEP and DEHP suggests considerable variability in exposure among adults to these two phthalates selected to represent two main daily activities: use of personal care products (dermal exposure to DEP) and diet (ingestion exposure to DEHP). We hypothesize that the patterns of exposure variability observed for DEP and DEHP will encompass those of other phthalates such as dibutyl and benzylbutyl phthalates, which do not have a clearly identified predominant pathway of exposure for the average adult person. However, additional research is needed to assess the variability in exposure to other phthalates and among populations that encompass different lifestyles and life span stages.

## Conclusions

When designing a biomonitoring study, one should consider the time of the sampling of the biological specimens (e.g., urine), particularly for phthalates and other nonpersistent compounds with short elimination t_1/2_ (i.e., a few hours). Our data suggest that for DEHP and, by extension, other compounds to which people are exposed mostly through diet, collecting 24-hr voids for only 1 day may not be advantageous for exposure assessment compared with collecting spot urine samples. On the other hand, if multiple urine collections are taken over a period of time, changing the time of day of collection may provide the most complete approach for exposure assessment, particularly when multiple phthalates and/or other compounds are evaluated, and thus minimize exposure misclassification. At the very least, we recommend that the time of day of urine collection and of the last urination be recorded. Despite the limitations resulting from the temporality of the biomarkers of nonpersistent chemicals such as phthalates, relying on biomonitoring urinary measures considerably strengthens the exposure assessment.

## Figures and Tables

**Table 1:** Urinary concentrations of MEP and MEHHP for all spot urine samples, first morning voids, and reconstructed 24-hr collections from all eight participants.^a^

	Urinary concentrations [uncorrected (μg/L) and creatinine corrected (μg/g)]								
	All spot samples (*n* = 427)			First morning void (*n* = 56)			Reconstructed 24-hr collection (*n* = 56)		
Phthalate metabolite	GM	Median	Interquartile	GM	Median	Interquartile	GM	Median	Interquartile
MEP									
μg/L	61.7	50.8	20–199	103	69	20.1–309	97.1	68.5	30.1–411
μg/g creatinine	105	75	32–280	86.7	45	30.3–161	136	97.1	41–409
MEHHP									
μg/L	22.2	21.3	6.7–71.6	40.2	36.5	22.9–73.9	38.8	28.4	16.5–115
μg/g creatinine	37.6	29.8	15.5–76.2	33.6	28.05	18.4–55.4	55.9	44.7	21.8–163

a For comparison purposes, the National Health and Nutrition Examination Survey (NHANES) 2005–2006 urinary concentrations (GM, median, 75th percentile) in micrograms per liter for adults are MEP (173, 168, 453) and MEHHP (23.4, 21.4, 48.6) (CDC 2010).

**Table 2:** Mean (± SD) urinary concentrations and variability of MEP for each study participant.^a^

	Mean MEP (μg/g creatinine)									
	Spot collections (including first morning void)							Days 1–7		
Participant	Monday	Tuesday	Wednesday	Thursday	Friday	Saturday	Sunday	Spot samples	First morning void	24-hr
P1	20.2 ± 7.1	33.1 ± 18.4	20.4 ± 15.9	13.0 ± 8.4	16.0 ± 11.3	23.2 ± 14.7	21.7 ± 6.5	20.6 ± 12.9	18.6 ± 5.1	21.3 ± 6.2
P2	160.7 ± 104.5	187.6 ± 110.2	97.7 ± 30.7	107.5 ± 52.7	220.2 ± 96.3	161.8 ± 111.6	87.5 ± 60.8	151.8 ± 95.2	93.5 ± 54.4	142.4 ± 53.1
P3	30.9 ± 14.9	67.4 ± 66.3	35.6 ± 16.0	36.3 ± 16.8	88.2 ± 60.1	28.0 ± 7.5	34.6 ± 21.1	45.3 ± 39.5	33.0 ± 7.4	39.6 ± 18.5
P4	1,641 ± 952	1,545 ± 1,317	2,980 ± 3,166	1,730 ± 1,136	2,631 ± 1,807	2,134 ± 1,478	3,174 ± 2,349	2,391 ± 2,065	4,848 ± 3,092	2,701 ± 1,283
P5	53.9 ± 14.1	101.4 ± 36.4	74.6 ± 55.3	41.6 ± 20.8	101.6 ± 118.4	53.6 ± 62.2	63.2 ± 47.6	69.3 ± 62.7	41.2 ± 25.2	60.4 ± 21.5
P6	305.8 ± 264.6	242.8 ± 217.2	165.6 ± 208.3	992.5 ± 1232.3	199.8 ± 165.6	242.9 ± 285.7	203.0 ± 108.4	341.1 ± 533.9	79.8 ± 41.4	334.7 ± 298.1
P7	49.4 ± 59.2	79.1 ± 64.7	102.7 ± 107.5	48.7 ± 21.9	41.3 ± 19.4	82.6 ± 73.4	56.5 ± 43.9	66.1 ± 62.1	35.4 ± 16.7	58.6 ± 20.4
P8	976.9 ± 892.4	736.1 ± 610.5	495.5 ± 345.6	363.9 ± 379.6	543.9 ± 445.3	364.9 ± 345.0	430.1 ± 413.6	586.1 ± 555.7	210.0 ± 44.9	554.5 ± 201.8

a The average intraperson CV of concentrations in the spot urine samples collected throughout the week was 92% and ranged from 62% (P1) to 157% (P6).

**Table 3:** Mean (± SD) urinary concentrations and variability of MEHHP for each study participant.^a^

	Mean MEHHP (μg/g creatinine)									
	Spot collections (including first morning void)							Days 1–7		
Participant	Monday	Tuesday	Wednesday	Thursday	Friday	Saturday	Sunday	Spot samples	First morning void	24-hr
P1	324.0 ± 296.1	163.4 ± 85.2	130.7 ± 162.2	373.2 ± 344.3	55.5 ± 54.3	24.4 ± 13.6	39.0 ± 52.6	158.0 ± 224.4	81.1 ± 56.7	139.9 ± 96.5
P2	26.6 ± 10.0	635.0 ± 730.9	67.6 ± 24.5	40.4 ± 26.6	33.8 ± 20.4	28.9 ± 12.6	126.5 ± 147.7	125.4 ± 329.8	47.7 ± 32.5	129.8 ± 139.7
P3	26.9 ± 19.9	273.4 ± 356.4	215.1 ± 235.6	473.5 ± 692.7	343.8 ± 415.3	66.5 ± 78.8	137.4 ± 123.1	218.5 ± 265.5	88.3 ± 116.7	182.2 ± 119.8
P4	16.5 ± 8.0	23.7 ± 16.6	20.1 ± 10.1	20.5 ± 8.6	18.7 ± 13.2	12.3 ± 6.4	136.5 ± 124.3	40.3 ± 68.9	15.2 ± 3.4	31.1 ± 32.9
P5	14.7 ± 5.9	12.9 ± 13.2	34.4 ± 20.3	25.2 ± 15.5	14.2 ± 10.8	18.5 ± 6.3	15.3 ± 12.9	19.0 ± 14.1	34.1 ± 9.3	24.0 ± 6.1
P6	95.7 ± 67.9	40.5 ± 14.0	33.5 ± 19.9	20.9 ± 6.9	19.8 ± 7.5	10.8 ± 6.2	25.3 ± 16.5	35.8 ± 37.1	30.1 ± 17.8	34.2 ± 24.0
P7	109.4 ± 118.0	26.7 ± 13.7	19.0 ± 5.4	19.1 ± 18.3	706.3 ± 603.4	107.0 ± 149.9	30.4 ± 29.8	109.0 ± 244.1	22.7 ± 8.2	143.6 ± 225.3
P8	8.3 ± 5.3	122.6 ± 131.6	337.8 ± 328.6	188.8 ± 137.0	243.7 ± 207.6	73.1 ± 61.4	16.7 ± 6.5	133.0 ± 187.2	60.5 ± 52.1	123.2 ± 105.0

a The average intraperson CV of concentrations in the spot urine samples collected throughout the week was 161% and ranged from 74% (P5) to 263% (P2).

**Table 4:** Effect of creatinine correction in the variance apportionment for the urinary concentrations of MEP and MEHHP in spot samples collected from eight persons over a period of 7 days.

	Creatinine unadjusted		Creatinine corrected		Creatinine as a covariate	
	Variance component	Percentage of total variance	Variance component	Percentage of total variance	Variance component	Percentage of total variance
MEP						

AIC	543.7		342.6		335.4	
Between persons	0.35	64	0.41	77	0.39	76
Within person, between days	0.01	2	0.01	2	0.01	2
Within person, within day	0.19	35	0.11	21	0.11	22

MEHHP						

AIC	820.3		579		574.5	
Between persons	0.1	18	0.06	17	0.06	17
Within person, between days	0.13	24	0.11	32	0.11	32
Within person, within day	0.32	58	0.17	51	0.17	51

**Table 5:** Variance apportionment for the creatinine-corrected concentrations of MEP and MEHHP in urine samples collected from eight persons over a 1-week period.

	Spot samples (*n* = 247)		First morning void (*n* = 56)		Reconstructed 24-hr voids (*n* = 56)	
Variance parameter	Variance component (95% CI)	Percentage of total variance^a^	Variance component (95% CI)	Percentage of total variance^a^	Variance component (95% CI)	Percentage of total variance^a^
MEP						

Between persons	0.41 (0.18–1.71)	77	0.53 (0.23–2.26)	91	0.47 (0.2–1.97)	94
Within person, between days	0.01 (0.003–0.04)	2	0.051 (0.04–0.08)	9	0.029 (0.02–0.045)	6
Within person, within day	0.11 (0.10–0.13)	21				

MEHHP						

Between persons	0.06 (0.02–0.48)	17	0.03 (0.01–0.33)	25	0.072 (0.026–0.59)	31
Within person, between days	0.11 (0.07–0.20)	32	0.09 (0.06–0.14)	75	0.16 (0.11–0.25)	69
Within person, within day	0.17 (0.15–0.20)	51				

a The ICC is the between-person percentage of total variance divided by 100.
